# The Routledge handbook of second language acquisition and technology

**DOI:** 10.3389/fpsyg.2023.1224826

**Published:** 2023-07-06

**Authors:** Fei Li, Huan Li

**Affiliations:** Department of English, School of Foreign Languages, China University of Geosciences, Wuhan, Hubei, China

**Keywords:** technology, SLA, research, intersection of technology and SLA, practice

As technology is increasingly important and applied in Second Language Acquisition (SLA), researchers and teachers alike have become more and more interested in understanding how technology could empower SLA theories, pedagogy, and research. To this end, the volume entitled The Routledge Handbook of Second Language Acquisition and Technology edited by Nicole Ziegler and Marta González-Lloret (Ziegler and González-Lloret, 2022) is a timely addition to address these needs. This book has brought together collections on the discussion and exploration of the effects of technology on second language (L2) learning by leading experts worldwide. Adopting different angles or approaches, this book not only introduces to readers the role of technology in L2 learning, but also serves as a valuable and useful resource to guide readers to deepen their understandings of the connections between technology and SLA, as well as providing teachers, students, researchers with comprehensive references for teaching, learning, and researching L2 in the context of technology-mediated SLA.

The volume falls into four parts, totalling 28 chapters. The topics were selected based on thematically and historically relevant theories, methodologies, and forthcoming advancements of SLA with technology. Part I (Chapters 1–8), “Theoretical and Methodological Perspectives”, overviews the application of technology in SLA, relevant theoretical frameworks, and different research methods to examine technology-enhanced language learning. Chapter 1 reviews the application of technology in SLA, highlights that the development of technology has advanced SLA research, and provides a look toward the future of research into technology and SLA respectively, preparing the readers for the following chapters. Chapters 2–8 expound on the methodologies and theoretical frameworks applicable to technology and SLA research from different aspects. More specifically, Chapter 2 provides guidance to narrow the gap between the theories of technology-mediated language teaching and its practice for teacher practitioners. Chapters 3–4 focus, respectively, on technology-mediated task-based language teaching (TBLT) and computer-mediated communication (CMC) from an interactionist perspective, unfolding how relevant technologies play a role in SLA. Chapters 5–7 examine topics from the advances and challenges underlying research methods across the borders of both technology and SLA, to applying Vygotskian theories to illustrate the use of technology-enhanced approaches in SLA process research, and to innovative methods such as big data to assist with the methodology and analysis of SLA research. Chapter 8 outlines the transformations technology has introduced to L2 assessments. On the one hand, these chapters explore how technology has advanced the SLA process and transformed methodologically the studies of its constructs. On the other hand, they present readers with how to apply the aforementioned paradigm to conduct research.

Part II (Chapters 9–16), “Developing Competences”, discusses research and applications of technology and specific language skills (including speaking in Chapter 11, listening in Chapter 10, reading in Chapter 13, writing in Chapter 14) and linguistic competence (i.e., pronunciation in Chapter12, vocabulary in Chapter 15, and pragmatics in Chapter 16) of L2 learners. Furthermore, some of the chapters go one step further to explore the various and concrete technology-assisted platforms and channels that can be used to enhance L2 learning. For example, Chapter 9 illustrates using chatbots for interactive dialogue communication to promote L2 learning while Chapter 11 probes into how oral communication could be facilitated through CMC. Each chapter in this part concentrates on only one aspect of L2 linguistic skill or competence: starting with reviewing previous research relevant to technology and its recent research trends, followed by exploring the influence that SLA theories have on technology use, closing the chapters with concrete design principles and cautions of technology-mediated education.

Part III (Chapters 17–24), “Spaces for Learning”, sheds light on how a diversity of digital spaces could facilitate SLA, with topics ranging from approaches to how online environment can be used to facilitate L2 learning (Chapter 17), how telecollaboration could be used and how its potential could be maximized in L2 learning (Chapter 18), how digital storytelling (DST) could improve different aspects of language learning for L2 learners (Chapter 19), how digital games and technology-mediated gamified learning environment could benefit learners effectively (Chapter 20), what multiplayer gaming implies for SLA research (Chapter 21), what technological advantages digital place-based learning have (Chapter 22), how interaction in virtual worlds (VWs) targeting language teaching could help to promote L2 learning as well as cultivate cross-cultural competence (Chapter 23), to how applications of mobile-assisted language learning (MALL) have evolved in the course of SLA research (Chapter 24). What distinguishes this part from the prior one is that instead of a focus on specific technological tools, it centers on broader technologies—digital space. Each chapter first overviews relevant theories and empirical research concerning the use of digital spaces in SLA, followed by specific application cases, ending with suggestions for new directions of pertinent research and advice on optimizing language teaching in practice. This part, together with the previous one, describes digital spaces from a macroscopical perspective and technological tools in SLA from a microscopical angle, respectively, thereby enabling readers both theoretically and practically to know how technology could enhance a particular aspect of linguistic competence and how to deepen their understanding of technologies in SLA.

As the major agents and a focal research theme of technology and SLA, learners, especially their individual differences could moderate technology-mediated L2 learning. Therefore, Chapters 25–27 in Part IV (“Focus on the Learner”) delve into how learner-related factors impact technology-mediated language learning, including age (Chapter 25), adult heritage language (Chapter 26), and learner autonomy (Chapter 27). More specifically, Chapter 25 points out that learners' learning experiences may vary to their different preferences of technologies; Chapter 26 concludes that the learning experiences of adult heritage language learners might play a role in their technology-mediated language learning. Both of these two chapters emphasize the effects of language experiences of L2 learners on their language learning. Chapter 27 advocates that active and critical interaction could ease the tension between learner autonomy and the pertaining technology. These three chapters not only inform readers of the significance of taking into account learner individual differences, but also put forward directions for further research. The last chapter (Chapter 28) explores the future trends and emerging prospects of integrating technology into language learning and proposes that the ever-renewing technology actualizes a more advanced and context-based language learning, echoing the first chapter in Part I thematically. As such, this closing chapter provides valuable references for readers to grasp the trends of technology and SLA research.

Compared with books on similar topics (e.g., Chapelle and Sauro, 2017), one of the strengths of the volume under discussion is its comprehensive contributions of research on technology and SLA. Not only does it introduce theories, methodologies, and practices of using technology in SLA, but also includes the impact technology exerts on SLA, as well as its historical origins, current situations, and future trends. Such comprehensiveness is beneficial for readers from various backgrounds. For L2 learners, illustrations of how technology can be used in L2 learning throughout many chapters of this book can inspire them to adopt appropriate technologies to improve their L2 learning outcomes. For teachers, the book's definition of key terms and description of theoretical development provide a theoretical basis and development status for them to understand how various technical tools can serve SLA. This could inspire teachers to hone technology-assisted pedagogical implementations. For researchers, especially novices, the content that showcases how technology might be employed in second language teaching and learning in many chapters could enlighten them to improve their research design.

Additionally, this book is characteristic of high readability, a typical feature shared by the Routledge handbook series. More specifically, each chapter follows a consistent pattern of content. It starts with a brief history of the topic discussed to arouse readers' interest, then illustrates the pertinent critical issues to provoke readers' thoughts, followed by an overview of current contributions to highlight landmark studies and major theories, and finally presents typical methodologies and future research directions for the topic, thus offering readers with expert guidance on how the area might be explored. The unified structure of all the chapters is useful for readers to focus quickly on contents that match their needs, thus freeing them from time to read less relevant information.

The handbook, of course, has its limitations. For experienced researchers, the comparatively succinct introduction of methodologies might be inadequate. If such readers intended to dig deeper into a particular topic, some extra reference books on research methods might be needed as complements. However, it is laudable that each chapter ends with a list of further reading, recommending books and papers that are worth reading for the given topic, which in some sense compromises the disadvantage. Therefore, the book is a comprehensive and systematic reference book with rich resources, deserving reading for researchers, teachers, and students who are interested in the intersection of technology and SLA research.

## Author contributions

FL: conceptualization, framework, draft, revision, supervision, and funding. HL: draft and revision. All authors contributed to the article and approved the submitted version.
